# Important Shapeshifter: Mechanisms Allowing Astrocytes to Respond to the Changing Nervous System During Development, Injury and Disease

**DOI:** 10.3389/fncel.2018.00261

**Published:** 2018-08-21

**Authors:** Juliane Schiweck, Britta J. Eickholt, Kai Murk

**Affiliations:** Institute for Biochemistry, Charité Universitätsmedizin Berlin, Berlin, Germany

**Keywords:** astrocytes, morphology, CNS, pathology, cytoskeleton, astrogliosis, brain trauma, synapse

## Abstract

Astrocytes are the most prevalent glial cells in the brain. Historically considered as “merely supporting” neurons, recent research has shown that astrocytes actively participate in a large variety of central nervous system (CNS) functions including synaptogenesis, neuronal transmission and synaptic plasticity. During disease and injury, astrocytes efficiently protect neurons by various means, notably by sealing them off from neurotoxic factors and repairing the blood-brain barrier. Their ramified morphology allows them to perform diverse tasks by interacting with synapses, blood vessels and other glial cells. In this review article, we provide an overview of how astrocytes acquire their complex morphology during development. We then move from the developing to the mature brain, and review current research on perisynaptic astrocytic processes, with a particular focus on how astrocytes engage synapses and modulate their formation and activity. Comprehensive changes have been reported in astrocyte cell shape in many CNS pathologies. Factors influencing these morphological changes are summarized in the context of brain pathologies, such as traumatic injury and degenerative conditions. We provide insight into the molecular, cellular and cytoskeletal machinery behind these shape changes which drive the dynamic remodeling in astrocyte morphology during injury and the development of pathologies.

## Introduction

Astrocytes have classically been depicted as star-like cells (Ramón y Cajal, 1913), however advanced visualization techniques have instead revealed astrocytes as bush- or sponge-like cells, each of which covers a distinct territory in the central nervous system (CNS; Bushong et al., 2004; Benediktsson et al., 2005). One of the most intriguing features of mature astrocytes is their extraordinary complexity: at high resolution, the ramification of the spongiform astrocytic processes into myriads of nanoscopic protrusions can be observed, frequently of sizes below the diffraction limit of light, and associated with synapses (Witcher et al., 2007; Medvedev et al., 2014). Their morphological complexity correlates with the plethora of functions astrocytes execute in the healthy CNS, including maintaining homeostasis, providing metabolic and neurotrophic support, promoting synaptogenesis, neurotransmitter uptake and recycling, modulating the plasticity and density of synapses. All of these tasks require close contacts between astrocytes and their targets, where the interactions are particularly plastic and can change depending on the individual physiological conditions. Moreover, astrocytes respond to pathologies in a process known as reactive astrogliosis, when they undergo substantial morphological changes to protect the CNS from inflammation, infection and neurodegeneration. In this review article, we summarize current knowledge of how astrocytes acquire, maintain and change their elaborate morphology through their molecular machinery and, in particular, via the cytoskeleton. We begin by describing the developmental process of astrogenesis and then focus on how astrocytes associate with and influence synapses in the mature CNS via their smallest processes, the perisynaptic astrocytic processes (PAPs). Finally, we discuss reactive astrogliosis and concentrate on the pathologies leading to the most profound shifts in astrocyte shape.

## Development: How to Become a Star

Like neurons, astrocytes originate from radial glial cells, which, despite their uniform appearance, form different progenitor domains within the ventricles of the developing brain, and generate cell subtypes with distinct morphological, functional and positional identities. Neurogenesis and gliogenesis from radial glial cells follow a step-like arrangement during development (Hirabayashi and Gotoh, 2005). In the mouse brain, neurogenesis starts at day E11 of prenatal development. The first cells exhibiting astrocyte characteristics appear between E16–E18, when neurogenesis decreases in favor of gliogenesis (Costa et al., 2009; Gao et al., 2014). However, the vast majority of astrocytes will only become detectable during the first 3 weeks after birth. Astrogenesis requires the early commitment of glial cell precursors to the astrocyte lineage, and the subsequent colonization of the CNS by differentiating astrocytes.

**Figure 1:**
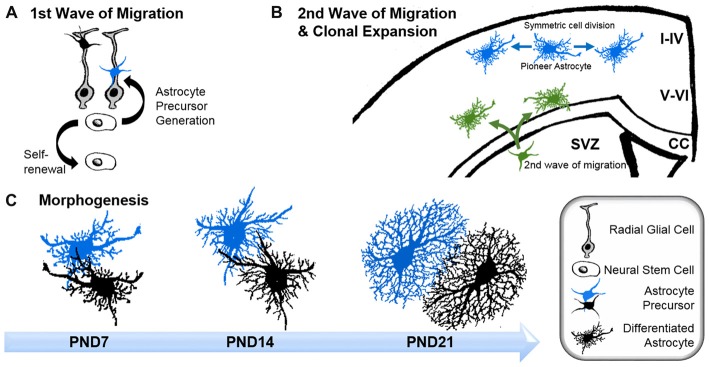
Astrogenesis and morphogenesis of astrocytes during development. **(A)** First wave of progenitor migration: asymmetric division of neural stem cells within the ventricular zones creates the first wave of astrocyte precursor cells (black and blue), which migrate along the processes of radial glial cells (gray) towards their final location in the central nervous system (CNS). **(B)** Clonal expansion of pioneering astrocytes and second wave of migrating astrocyte progenitor during late development: pioneering astrocytes of the first migration wave (blue) undergo symmetric cell division in the upper cortical layers, while a second wave of astrocyte progenitors emerges from the subventricular zone (SVZ; green), predominantly colonizing the lower cortical layers. Corpus callosum (CC). **(C)** Time course of astrocyte morphogenesis during postnatal development, demonstrated by two neighboring astrocytes (blue and black). Differentiating astrocytes possess long main processes at postnatal day (PND) 7, which invade the domains of neighboring astrocytes. At PND14, ramification into smaller processes has increased, whilst the extent of invasion into domains of neighboring astrocytes is reduced. Three weeks after birth, at PND21, astrocytes have acquired their complex morphologies within their distinct domains. At this stage, only very limited intermingling with neighboring astrocytes occurs.

Early and intermediate astrocyte progenitors are difficult to trace and manipulate. To our knowledge, unique factors which actively instruct precursors to differentiate into astrocytes have not to date been identified. Rather, it appears that astrogenesis relies on the repression of neurogenic genes through numerous signaling pathways (Kanski et al., 2014; Nagao et al., 2016). However, the pro-gliogenic transcription factors involved are not restricted to astrocyte differentiation as they are also required for the generation of oligodendrocytes (Stolt et al., 2003). To acquire their positional identity, differentiating astrocytes “re-use” molecular mechanisms, such as the homeodomain code, which neurons follow earlier in development (Hochstim et al., 2008). Subsequent to the principal commitment of precursor cells to the astrocyte lineage, astrocyte progenitors leave their cradles and populate the entire CNS (Bandeira et al., 2009). Region-specific fate mapping in the spinal cord and cortex revealed that the migration of astrocyte progenitors occurs along radial glial cell processes (Figure 1A; Tsai et al., 2012). However these processes disappear early in postnatal development, raising the question as to how later-appearing astrocytes reach their specific locations throughout the CNS (Rakic, 2003). One possible explanation is that early emerging astrocytic precursors migrate along the radial glial cells and pioneer unhabituated brain regions. After reaching their final positions, these pioneer astrocytes expand by symmetric cell division and colonize defined brain areas. Tracing experiments in the postnatal cortex demonstrated the capability of astrocytes to generate up to 50% of the total astrocyte numbers via symmetric cell division (Ge et al., 2012). Another 10% of cortical astrocytes are generated directly from radial glial cells during their final cell division (Masahira et al., 2006). The remaining 40% of the total astrocyte population are derived from progenitor cells originating from the subventricular zone (SVZ). Despite the absence of the guiding processes of radial glial cells, this astrocyte population nonetheless continues to migrate along the tracks of pioneering cells (Jacobsen and Miller, 2003; Rakic, 2003). It is currently unknown how these “latecomers” move along formerly beaten tracks, nor how they find their precise destination in the developing brain. These “second wave” astrocytes typically cover shorter distances compared to the pioneering astrocytes, and populate the cortical layers adjacent to the SVZs (Figure 1B; Ge et al., 2012).

After arriving at their designated position, astrocytes begin to express their canonical markers, such as glial fibrillary acidic protein (GFAP), S100β, Aldh1L1, Sox9, AldoC, Glt1 and glutamine synthase (for a review see Molofsky et al., 2012). During this period, astrocytes also initiate the formation of their stellate morphology (Figure 1C). The time course in forming the uniquely ramified morphology of astrocytes has been documented in rats by Bushong et al. (2004) using immunolabeling and dye filling. In brief, during the first week of postnatal development, protoplasmic astrocytes display considerable diversity in morphology. S100ß and GFAP-positive cells extend between three and six long main processes and develop a ramification of fine, stringy or filamentous processes. At this stage, astrocytes are not yet restricted to the defined domains typically observed for mature astrocytes, and can extend their long main processes beyond any rudimentary boundaries. By postnatal week 2, astrocytes have adopted a more uniform morphology, with complex ramification patterns of their processes. However, the astrocytic processes are still stringy or filiform and have not yet matured into the characteristic spongiform shapes. During this period, the well-documented astrocytic territorial domains are recognizable for the first time, although overlap of processes is still present. By postnatal week 3–4, astrocytes have matured into their terminally differentiated state through the establishment of dense spongiform processes within their individual domains. However, the morphological and functional identity of astrocytes is not entirely hardwired but rather depends on the individual environment, particularly on the input of surrounding neurons throughout their lifetime.

Scientists are only now beginning to comprehend the molecular signals and their downstream mechanisms involved in establishing astrocyte morphology. Genetic studies in *Drosophila* identified neuron-secreted FGFs as critical for the elaboration of astrocyte morphology (Stork et al., 2014). Recent work in the optical lobe of *Drosophila* showed a role for the transmembrane leucine-rich repeat protein Lapsyn in regulating the morphogenesis of the astrocyte-like medulla neuropil glia, which also cooperates with FGF to promote astrocyte branch formation and survival (Richier et al., 2017). Moreover, a comprehensive study in the rodent cerebellum indicated that the acquired identity of astrocytes could be overwritten by surrounding neurons (Farmer et al., 2016). In this study, the authors demonstrate that the morphogen sonic hedgehog secreted from the adult Purkinje cells sustains the functional identity of adjacent Bergmann glia. Manipulating sonic hedgehog in the mature cerebellum induces another astrocyte subpopulation, so-called velate astrocytes, to acquire the transcriptome and electrophysiological characteristics of Bergmann glia. Taken together, these studies show that astrocytes represent a population of particularly plastic and heterogeneous cells, which are responsive to their neuronal neighbors.

## What Stars Are Made of and What Keeps Them in Shape

The development of the stunning complexity of astrocytes from the thin precursor cylindrical radial glial cells necessarily involves extensive remodeling of the cytoskeleton. However very little is known about the molecular machinery behind these comprehensive shapeshifts. A key reason contributing to this limited insight is that the commonly-used cell culture astrocyte models do not accurately recapitulate astrocytes *in situ* or during normal morphogenesis. While neurons develop spontaneously from apolar progenitors and form mature cells with elaborate axon and dendrite morphologies in culture, astrocytes in serum-enriched cultures acquire a polygonal morphology analogous to non-neuronal cells (McCarthy and de Velllis, 1980). More importantly, transcriptome analysis revealed that cultured polygonal astrocytes have different genetic profiles compared to astrocytes *in situ*, but share similar profiles with immature and reactive astrocytes (Foo et al., 2012). Changes in culture conditions and pharmacological treatments, such as artificially increasing intracellular cAMP levels, can convert polygonal astrocytes into stellate astrocytes within a matter of minutes (Shapiro, 1973). However, this process does not resemble *in vivo* differentiation of progenitor cells into ramified astrocytes. Instead, this so-called process of “stellation” is a model used to identify the cellular architecture necessary to maintain the typical astrocyte morphology. Keeping these limitations in mind, we review here what is known about the cytoskeletal organization of stellate astrocytes in culture and *in vivo*.

### Microtubules

The function of microtubules has rarely been addressed in stellate astrocytes in general, and during astrogenesis in particular. An early electron microscopy study demonstrated dense microtubule networks in mature astrocytes, compared to immature astrocytes that exhibit more loosely packed microtubules (Peters and Vaughn, 1967). Only recently, microtubules were visualized for the first time in radial glia and their astroglia progeny in living brain tissue (Eom et al., 2011). Short-term live imaging revealed the restriction of microtubules to the main processes, where they appear to be relatively stable. In cell culture, the *in vivo* distribution of microtubules is analogously present in stellating astrocytes, where microtubules co-extend with intermediate filaments in forming the processes. Pharmacological inhibition of microtubule polymerization during stellation prevents the transition of astrocytes into the star-like cells (Goetschy et al., 1986). Nonetheless comprehensive analyses of the changes and functions of microtubules in astrocytes during stellation are not currently available and further studies are needed.

### Intermediate Filaments

**Figure 2:**
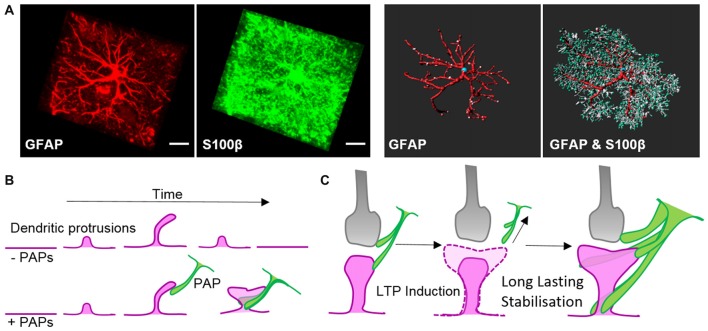
Morphology of astrocytes *in vivo* and their dynamic association with synapses. **(A)** *Z*-projection of a cortical rat astrocyte (P14), stained for glial fibrillary acidic protein (GFAP; red) and S100β (green), by confocal microscopy after tissue clearance (left). Scale bars: 10 μm. 3D rendering and morphometric analyses show the restriction of the intermediate filament protein GFAP to main processes, which are decorated with myriads of fine S100β-positive processes (right; with permission from Murk et al., 2013). **(B)** Schematic of dendritic filopodia, with the precursors of dendritic spines, emerging from dendrites (magenta) in the absence (top) or presence of perisynaptic astrocytic processes (PAPs; green, bottom). Without the support of astrocytic processes, sprouting dendritic filopodia have a short lifespan and are likely to retract. Astrocytic processes contact filopodia-like protrusions, which then exhibit an increased stability and higher tendency to develop into mature dendritic spines. **(C)** Schematic of a mature tripartite synapse consisting of the pre-synapse (gray), post-synapse (magenta) and PAPs (green), which respond to long-term potentiation (LTP) with structural changes. Induction of LTP transiently enhances the motility and retraction of PAPs allowing growth of the postsynaptic dendritic spine to occur. Subsequent to dendritic spine remodeling, PAPs intensify their coverage of synapses.

Intermediate filaments have been studied extensively in astrocytes thanks to the availability of knockout mouse models. Like microtubules, intermediate filaments are restricted to the main processes of astrocytes *in vivo* (Figure 2A; Bushong et al., 2002). The interconnected scaffold-like network in astrocytes is a composite of different intermediate filament proteins which change during development and maturation. Astrocyte progenitors express the intermediate filament proteins of vimentin, nestin and synemin, whereas maturing and differentiated astrocytes express only GFAP and vimentin (Sultana et al., 2000). During adulthood, GFAP expression in astrocytes varies largely depending on the brain region. In the cortex, 85% of astrocytes are negative for GFAP but upregulate this intermediate filament protein again during aging (Kimelberg, 2004). GFAP or vimentin are the essential subunits for polymerizing intermediate filaments. In immature astrocytes deficient in both GFAP and vimentin, nestin itself is unable to polymerize into filaments (Pekny et al., 1999). Intermediate filaments also play a scaffolding role in organizing the cytoplasm and organelles, and modulate directed vesicle transport (Potokar et al., 2007). However, deficiencies in GFAP and/or vimentin have no obvious effect on the outgrowth of astrocytic processes in mixed neuronal cultures or on the development and maturation of astrocytes *in vivo*, despite impaired intermediate filament formation (Pekny et al., 1995, 1998).

### The Actin Cytoskeleton

Studies that induced drastic shapeshifting between polygonal and stellate morphologies of astrocytes in cell culture have increased our understanding of the actin cytoskeleton in this process. In their polygonal shape, astrocytes possess prominent actin fibers that depend on a high activation state of myosin-II (John, 2004). In contrast, when astrocytes adopt stellate shapes *in vitro*, they exhibit a lack of actin fibers and instead establish Arp2/3-dependent actin networks (Murk et al., 2013). Branched Arp2/3-based actin arrays and the machinery for linear actin filaments act in direct opposition to each other, and rely on distinct signaling pathways (Rotty et al., 2015). Signaling through receptors such as beta-adrenergic receptors activates PKA and/or PKCepsilon and inhibits the ROCK-RhoA-axis. Concurrently, an increase in Rac1 activity drives the remodeling of contractile actin fibers into branched actin arrays (Moonen et al., 1976; Ramakers and Moolenaar, 1998; Burgos et al., 2007; Racchetti et al., 2012; Kobayashi et al., 2013; Murk et al., 2013). In addition to experiments using polygonal astrocytes, improved cell culture models based on defined culture media (Scholze et al., 2014; Wolfes and Dean, 2018), immunopanning (Foo et al., 2012), 3D scaffolds and matrices (Lau et al., 2014; Woo et al., 2017), and organoids (Renner et al., 2017) will facilitate functional studies of the underlying molecular machinery controlling astrocyte morphogenesis.

## A Starring Role for Astrocytes at Synapses

The main processes of astrocytes form the basis of their star-like shape, while their true spongiform morphology relies on myriads of small protrusions. After birth, the sophisticated ramification into nanoscopic astroglial protrusions occurs in parallel with synaptogenesis, when astrocytes begin to associate with developing neuronal connections via PAPs and establish “tripartite synapses.” The frequency of establishing tripartite synapses depends on the brain area and local neuronal activity (Araque et al., 1999). Studies in different organisms from *Drosophila* to humans indicate PAPs as conserved structures that are essential for brain function. Consistently, the number of PAP-associated synapses covered by astrocytes is increasing from fly to human (Oberheim et al., 2009; Stork et al., 2014). For instance, a single rodent astrocyte can associate with up to 120,000 synapses (Bushong et al., 2002), whereas a human astrocyte, according to a several-fold larger cell size and a 10-fold increase in the number of main processes, contacts up to two million synapses (Oberheim et al., 2006, 2009). These hominid-specific characteristics in astrocyte morphology are fundamental for sophisticated learning and memory, as shown in chimeric mice harboring human induced pluripotent stem cells (iPSC)-derived astrocytes. The transplanted human astrocytes replace the host’s astroglia and develop the characteristic larger ramification of normal human astrocytes. Moreover, the chimeric mice show substantially enhanced synaptic plasticity and learning, compared to control animals (Han et al., 2013). Although further experimental evidence is needed to directly test for the link of PAPs in moderating synaptic responses in tripartite synapses, the correlation of enhanced learning and memory with increasing ramification and astrocyte-synapse interactions is impressive. It implies PAPs are key structures that astrocytes require to actively partake in synaptic activity.

Astrocytes control the ionic homeostasis of the CNS (Simard and Nedergaard, 2004) where they modulate synaptic plasticity by active buffering of potassium ions (Pannasch et al., 2011). In addition, astrocytes clear neurotransmitters and recycle their inactive derivatives back to the presynaptic terminal (Schousboe et al., 2013). Gliotransmitters such as d-serine, glutamate, ATP, taurine and TNFα are secreted by astrocytes and regulate synaptic activity (Panatier et al., 2006; Stellwagen and Malenka, 2006; Cao et al., 2013; Martin-Fernandez et al., 2017; Tan et al., 2017; Van Horn et al., 2017). During postnatal development, astrocytes sequentially secrete a range of factors, such as thrombospondins and hevin, that are involved in initiating the formation of silent synapses (Christopherson et al., 2005; Kucukdereli et al., 2011; Singh et al., 2016). Subsequently, other astrocyte-secreted factors, such as glypicans 4 and 6, turn the established silent synapses into active connections (Allen et al., 2012). To establish and maintain a neuronal network with individually controllable synapses through diffusible molecules require a tight spatial-temporal control of signaling in vicinity of synapses. The molecular equipment required for these tasks, including metabotropic glutamate receptors (mGluRs), glutamate transporters and ion channels, is enriched in PAPs and is thus in immediate proximity to synapses (Chaudhry et al., 1995; Higashi et al., 2001; Lavialle et al., 2011).

Along with locally secreted factors, PAPs influence synapses through direct contact mediated by cell adhesion molecules. Filopodial precursors of dendritic spines are endowed with a substantially enhanced lifespan and develop into mature dendritic spines with greater frequency in direct association with PAPs (Figure 2B, Nishida and Okabe, 2007). Immunohistochemical and electron microscopy studies showed several cell adhesion molecules that appear to link PAPs to synapses. Several molecules, such as SynCAM1, NCAM and α_v_β_3_-integrins, form connections between PAPs and synapses (Theodosis et al., 2004; Hermosilla et al., 2008; Sandau et al., 2011), but their true nature regarding astrocyte-neuron interactions still needs to be demonstrated in functional approaches. One striking example of a shapeshifter transmembrane protein on astrocytes that affects neuronal functions is neuroligin 2. Neuroligin 2 localizes to astrocyte processes and its specific deletion in astrocytes affects astrocyte morphogenesis and substantially imbalances neuronal circuits by impairing the formation of excitatory synapses (Stogsdill et al., 2017). The γ-protocadherins (γ-Pcdhs) are another candidate for cell adhesion molecules that can functionally connect astrocytes and neurons (Keeler et al., 2015). γ-Pcdhs are present on PAPs and directly drive synaptogenesis in neuron-astrocyte co-culture models (Garrett and Weiner, 2009). However, γ-Pcdhs also directly link neuronal pre- and post-synapses (Rubinstein et al., 2015; Molumby et al., 2016, 2017). Accordingly, the modulation of synapses via γ-Pcdhs likely only relies on PAPs to some extent.

Several studies indicate a supportive role for astrocytes on synapses, whereas others discovered an essential function in reducing the total number and structural plasticity of synapses. One of the first pairs of cell adhesion molecules analyzed in a functional approach is the receptor tyrosine kinase EphA4 and its ligand ephrin-A3 that exhibit a negative regulative effect on excitatory synapses. In the adult hippocampus, EphA4 is restricted to dendritic spines, whereas ephrin-A3 is enriched on adjacent PAPs. The interaction of neuronal EphA4 with astrocytic ephrin-A3 evokes spine retraction, a process which is distorted in EphA4-deficient mice (Murai et al., 2003). Reverse signaling from neuronal EphA4 towards astrocytic ephrin-A3 evokes decreased levels of the glutamate transporters Glt1 and GLAST in PAPs, which correlates with shrinking dendritic spines (Carmona et al., 2009; Filosa et al., 2014). EphA4-ephrin-A3 signaling thus represents a means of how cell-cell contact-based neuron-glial communication can induce negative structural plasticity in neurons. In addition, astrocytic processes contain the phagocytic receptors MERKT and MEGF10 that have both been identified in mediating synapse elimination through active engulfment (Chung et al., 2013).

### The Dynamic Cytoskeleton in PAPs

The interplay of PAPs with synapses alters depending on the organism’s physiological condition, such as parturition, lactation, chronic dehydration, starvation, voluntary exercise or sleep deprivation (Theodosis, 2002; Procko et al., 2011; Tatsumi et al., 2016; Bellesi et al., 2017). Recently, quantitative measurements in the sensory-visual cortex during eye occlusion demonstrated the plasticity of PAPs covering synapses. These experiments demonstrated clearly that PAPs are particularly dynamic during development as well as during activity of synaptic circuits (Stogsdill et al., 2017). Overall morphological changes in PAPs in response to environmental cues seem to occur slowly over hours to days, whereas live imaging experiments *ex* and *in vivo* revealed extensive structural plasticity PAPs within much shorter time-frames—of only minutes (Figure 2C; Bernardinelli et al., 2014; Perez-Alvarez et al., 2014). Activation of synapses through stimulating metabotropic glutamate receptors or glutamate uncaging triggered transiently increased PAP motility which ultimately evoked a more stable interaction of PAPs with dendritic spines (Figure 2C; Bernardinelli et al., 2014).

Rapid changes in PAPs highlight a prominent role for a dynamic cytoskeleton during structural remodeling of astrocytes (Bernardinelli et al., 2014). In view of the fact that PAPs are devoid of microtubules and intermediate filaments, all structural changes in PAPs are likely to involve the reorganization of the actin cytoskeleton. Dynamic PAPs present as miniature versions of lamellipodia and filopodia, the F-actin-rich subcellular compartments of migrating non-neuronal cells (Hirrlinger et al., 2004). Analogous to typical lamellipodia, extensions of PAPs are severely impaired upon inactivating the small GTPase Rac1 (Nishida and Okabe, 2007), and likely depend on inhibition of Rac1 downstream targets, such as actin regulators forming Arp2/3-dependent branched actin arrays (Rottner et al., 2017). Accordingly, direct inhibition of the Arp2/3 complex in brain tissue or knockdown of its upstream regulators N-WASP, WAVE2 and PICK1 in cell culture induces profound alterations in the morphological complexity of astrocytes. On the one hand, Arp2/3 inactivation *in situ* has been associated with the loss of fine astrocytic processes (Murk et al., 2013). On the other hand, small G-actin binding proteins called profilins, which charge actin monomers with ATP and mainly enhance actin polymerization (Jockusch et al., 2007), modulate the overall complexity of astrocytes and actin turnover in PAPs. Isoform-specific knockdowns of either ubiquitous profilin 1 or CNS-specific profilin 2a which is involved in neurons in presynaptic membrane trafficking and dendritic spine remodeling (Pilo Boyl et al., 2007; Michaelsen et al., 2010), reduce the total volume of astrocytes in organotypic slices. However, selective inhibition of profilin 1 affects the number and movement of filopodia processes by slowing the reorganization of filamentous actin (Molotkov et al., 2013; Schweinhuber et al., 2015).

Another actin binding protein prominently localized to PAPs is ezrin, a linker protein connecting the actin cytoskeleton directly to the plasma membrane (Derouiche and Frotscher, 2001; Haseleu et al., 2013). Active ezrin is exclusively located in PAPs and is required for motility of astrocytic filopodia induced by glutamate-activating mGluR3 and 5 (Lavialle et al., 2011). In addition to the typical actin regulators, connexin30 has also been shown to regulate synaptic strength by controlling the synaptic location of astroglial processes (Pannasch et al., 2014). Deletion of connexin30 evokes increased ramification and process length, with PAPs invading the synaptic cleft and causing elevated uptake of glutamate at excitatory synapses. The molecular details that underlie connexin30 regulation of PAPs are currently unclear, but appear to involve its intracellular C-terminus, which is likely to be a hub for interactions with as-yet unidentified actin regulators. A potential candidate might be drebrin, an actin regulator binding sidewise to filaments, which has been shown to interact with connexin43 in cultured astrocytes (Butkevich et al., 2004). However, whether drebrin does indeed bind and control connexin30 function in astrocytes is not known.

## Stars With Sensitive Feet

While astrocytes use PAPs to register, support and modulate neuronal activity at synapses, they also almost entirely encompass the vasculature of the CNS with processes known as endfeet. In conjunction with specialized endothelial cells, pericytes and an elaborate basal lamina, astrocytic endfeet create the blood brain barrier to facilitate the brain’s selective uptake of required nutrients and metabolites, the exclusion of toxic substances and immune cells as well as the efflux of waste products (Daneman and Prat, 2015). Perivascular endfeet possess prominent orthogonal arrays of intramembranous particles at their plasma membrane, where aquaporins and potassium channels accumulate (Rash et al., 1998; Warth et al., 2005). Accordingly, astrocytic endfeet act as major hubs to regulate the ion and water homeostasis in the CNS (Min and van der Knaap, 2018). Endfeet and PAPs emplace astrocytes as cellular interface between synapses and vasculature, where they enable the appropriate supply of oxygen and energy to neurons according to their activity-dependent demands. Intensive research showed the ability of astrocytes to regulate the local cerebral blood flow by sensing and relaying neuronal signals to the vasculature. The currently discussed mechanisms of astrocyte-mediated blood flow control comprise potassium siphoning, metabolic neurovascular coupling and intracellular calcium waves, which evoke the synthesis of vasoactive metabolites (see for review MacVicar and Newman, 2015). Despite the relevance of endfeet for astrocyte functions, very little information is available on their intracellular structures. Microtubules have been visualized in endfeet of perivascular astrocytes in a single *in vivo* study but their role in this subcellular compartment is unknown (Eom et al., 2011). Immunohistochemical analyses revealed prominent GFAP-positive intermediate filaments in endfeet. Endfeet differ distinctively in their fine structures between rodents and humans. Perivascular GFAP appears in rats as rosettes around blood vessels and creates the impression of an incomplete coverage of the vasculature by the astrocyte endfeet (Rungger-Brändle et al., 1993). In contrast, in humans GFAP in endfeet exhibits a densely packed, tile-like pattern entirely encompassing the blood vessels (Oberheim et al., 2009). More recent electron microscopy 3D reconstructions demonstrated the complete coverage of blood vessels in the rat brain by astrocyte endfeet despite the rosette-like GFAP localization (Mathiisen et al., 2010). Under normal conditions, blood vessels in the brain and spinal cord from GFAP- and vimentin-deficient mice frequently show increased dilatation (Pekny et al., 1999). To our knowledge, the microanatomy of actin filaments in astrocyte endfeet is unknown. Indications for a putative role of actin in astrocytes are indirect, reported from loss-of-function studies and cell culture experiments. Astrocyte endfeet have enriched protein complexes composed of the transmembrane protein dystroglycan and its associated ligands syntrophin and dystrophin, and in other cell types this complex bridges the actin cytoskeleton with laminins in the extracellular matrix (Higginson and Winder, 2005). Moreover, the defined localization of potassium channels and aquaporin-4 in the plasma membrane of endfeet partially depends on tethered dystroglycan protein complexes. In cell culture, the actin cytoskeleton directly governs the localization of aquaporin-4 (Nicchia et al., 2008). Deleting the focal adhesion adapter protein vinculin in Bergmann glia disturbs the GFAP distribution in endfeet but has no obvious effect on neurovascular functions (Winkler et al., 2013).

## From Stars to Scars

### Dealing With Insults

**Figure 3:**
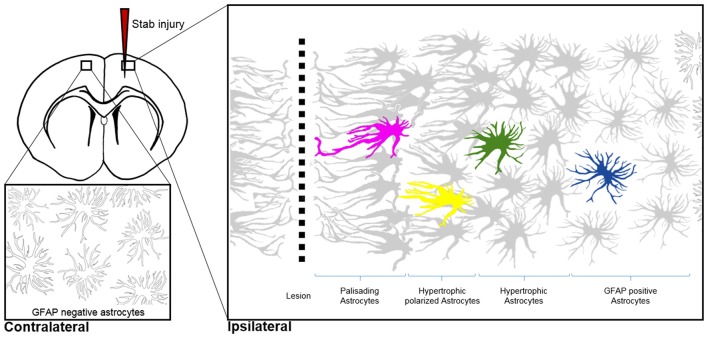
Morphological hallmarks of reactive astrocytes in traumatic brain injury. Typical distribution of reactive astrocytes after a cortical stab wound in the mouse cortex 7 days after injury. Schematic drawings show the main processes of astrocytes, which contain high levels of GFAP (gray) upon injury. GFAP-positive astrocytes exhibit different morphologies depending on their distance from the lesion site: in the immediate vicinity (magenta), astrocytes polarize and extend long “palisading” processes. In the second row, multiple processes of the hypertrophic astrocytes (yellow) are orientated towards the injury site; with increased distance, astrocytes possess hypertrophic cell bodies and primarily main processes (green). Finally, reactive astrocytes at the border of the unaffected parenchyma upregulate GFAP but show no signs of hypertrophy (blue). Note that cortical astrocytes in the uninjured contralateral hemisphere sparsely express GFAP, as indicated with silhouettes (bottom left image).

Under normal conditions, the majority of astroglia occupy a distinct territory in the brain parenchyma, with the most distant processes of neighboring astrocytes exhibiting very limited intermingling (Bushong et al., 2002). In terms of morphological plasticity, microscopic observations at low magnification give the impression that astrocytes are rather quiescent under normal conditions. Only the smallest astrocyte protrusions—such as PAPs—are motile and alter their shape upon neuronal activity. However, in response to injury and other pathological conditions, astrocytes undergo astrogliosis and become “reactive.” This process is accompanied by dramatic changes in morphology, including prominent hypertrophy, altered ramification and outgrowth of particularly long processes (Figure 3). Astrogliosis is recognized as a defense mechanism that controls inflammation and the blood-brain barrier integrity. An important contribution of astrogliosis is the isolation of non-injured tissue from damaged areas, and the support of neuronal circuit and tissue regeneration (Burda et al., 2016). The molecular triggers and specific signaling mechanisms of reactive astrogliosis have been reviewed in detail (Sofroniew, 2009; Ben Haim et al., 2015); here we discuss known morphological changes that occur during reactive astrogliosis and present the scope of their physiological function.

Shapeshifting of reactive astrocytes depends on the nature and severity of the CNS insult. Acute and diffuse trauma without tissue damage evoke a transient upregulation of GFAP and other intermediate filament proteins in conjunction with minor and reversible hypertrophy of both astrocyte cell bodies and main processes (Wilhelmsson et al., 2006). Reactive astrocytes thereby uphold their local domains and do not proliferate. Longer lasting and more severe injuries provoke higher GFAP levels in astrocytes and lead to more prominent cell body hypertrophy associated with the interpenetrative extension of distant processes into adjacent astrocyte domains and occasional hot spots of cell proliferation (Myer et al., 2006). Reverting hypertrophy and downregulating GFAP levels in highly reactive astrocytes is possible, but correlates negatively with the duration and severity of the diffuse brain injury (Petito et al., 1990). Traumatic brain injuries with severe tissue penetration and profound lesions create the most prominent response of reactive astrocytes by collectively forming a glial scar to protect the surrounding parenchyma from spreading inflammation, infection and neurodegeneration (Figure 3). Astrocytes that are in the vicinity of focal lesions resemble “palisades,” bordering, orienting and extending long processes towards the injury site. Live imaging experiments in mice with cortical stab wounds revealed that 45% of reactive astrocytes are polarized. Polarized and elongated cells originate from mature astrocytes through their transient de-differentiation and proliferation (Bardehle et al., 2013; Wanner et al., 2013). Astrocytes behind the palisades manifest prominent hypertrophy and a tendency to orientate most processes towards the lesion (Kanemaru et al., 2013). With increasing distance from the injury, the hallmarks of astrogliosis—such as hypertrophy and elevated GFAP levels—tend to decrease, and appear to be analogous to mild diffuse CNS injuries (Figure 3). Live imaging and ablation experiments demonstrated the local occurrence of reactive astrogliosis at the insult site without any active migration of additional astrocytes from neighboring brain areas (Bardehle et al., 2013; Tsai et al., 2012).

In comparison to microglia that respond to CNS insults within minutes, the time course of morphological changes for astrocytes is relatively slow (Nimmerjahn et al., 2005). Hypertrophy and GFAP upregulation appear after 2–3 days post-injury, with palisading astrocytes observed approximately 1 week after injury (Robel et al., 2011). Nevertheless, astrogliosis and glial scarring are essential for the early phase of tissue protection after traumatic injuries, as reactive astrocytes protect against spreading cell death and inflammation after spinal cord injuries (Faulkner et al., 2004). In close proximity to lesion sites, palisading and hypertrophic astrocytes sustain their reactivity permanently, whereas astrocytes at greater distances from the injury return to their normal state (Bardehle et al., 2013). Persistent glial scars have been considered a crucial part of the failure of regenerative treatments of traumatic brain and spinal cord injuries (Cregg et al., 2014). Whether reactive astrocytes represent a major hurdle or a benefit for regenerative treatments remains a topic of intensive debate (Anderson et al., 2016).

### Stars in Disease

Scar-forming astrocytes have been reported in a number of pathological conditions such as Alzheimer’s disease (AD) and brain tumors, but follow a more complex response pattern during the progression of these diseases. During the later phases of AD, reactive astrocytes form prominent glial scar-like barriers around amyloid plaques and disrupt the anthropoid-specific architecture of non-reactive astrocytes in the neocortex (Colombo et al., 2002). Whether astrocyte reactivity also occurs in earlier phases of AD is currently under debate. PET scans in patients and some mouse models indicate astrogliosis as an early component of AD development (Heneka et al., 2005; Carter et al., 2012). In contrast, the triple transgenic mouse model of AD exhibits comprehensive cytoskeletal atrophy of astrocytes prior to amyloid plaque-associated astrogliosis (Kulijewicz-Nawrot et al., 2012). The precise role of astrocytes in AD needs to be further investigated as both astrocyte atrophy and astrogliosis may indicate the participation of astrocytes in the neuropathology of this disease.

**Figure 4:**
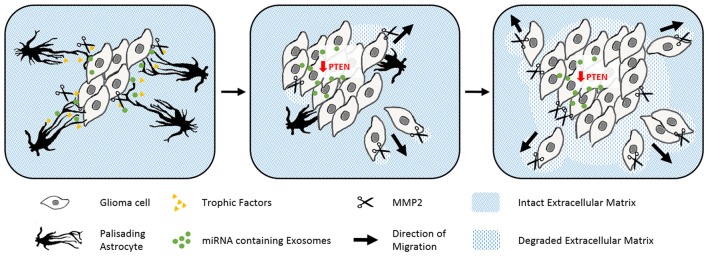
The abuse of scarring astrocytes by glioma. Schematic drawings of how reactive astrocytes turn from initially tumor containing to cancer promoting cells. Astrocytes (black) form prominent palisading processes in response to growing glioma masses (gray rhomboid cells). Glioma derived signals force the reactive astrocytes to secret trophic factors (yellow), pro matrix metalloproteinase-2 (scissors) and microRNA containing exosomes (green, left). The uptake of microRNA containing exosomes evokes the epigenetical downregulation of tumor suppressing PTEN in glioma. PTEN depletion and astrocyte-derived trophic signals enhance the cell proliferation of the glioma cells. Expanding tumor cells use matrix metalloproteinases to degrade the surrounding extracellular matrix (blue stripped pattern) and begin to penetrate the glial scar (center). In the late phase, glioma have comprehensively degraded their adjacent extracellular matrix and seemingly consumed the originally tumor encompassing astrocytes (right).

Gliomas are also surrounded by activated astrocytes initially with prominent palisades (Figure 4; Le et al., 2003). However in this disease, the astrogliosis response is unable to isolate the tumor from the intact tissue. Gliomas exploit reactive astrocytes together with other cell types to create a favorable microenvironment. Neurotrophic factors and pro-matrix metalloproteinase-2 (MMP2) secreted from astrocytes promote tumor growth (Hoelzinger et al., 2007; Lee et al., 2009). Interestingly, gliomas enhance cell proliferation and invasive migration via an astrocyte-induced knockdown of the key tumor suppressor PTEN. The epigenetic downregulation of PTEN in the cancer cells relies on the uptake of microRNAs, which are packaged in exosomes and secreted from neighboring astrocytes upon tumor-derived signals (Zhang et al., 2015). Brain tumors then consume the bordering reactive astrocytes over time, before they invade and disrupt the surrounding tissue as well as the blood-brain barrier (Watkins et al., 2014).

Profound changes in astrocyte cell morphology and function are induced by external signals and computed from pathological and physiological cues. Non-reactive astrocytes persistently survey their environment for abnormalities such as pathogenic, aggregated and serum proteins, as well as cytokines and chemokines. Contact with these cues activates multiple receptors and signaling pathways, which then trigger astrocyte reactivity programs (Burda et al., 2016). Besides pathological signals, astrocytes constantly receive instructions from healthy neurons, which actively suppress astrocyte reactivity. Key molecules within these signaling pathways include neuron-derived FGF-2 and astrocytic β1-integrin. Interestingly, loss-of-function of both factors causes astrocyte reactivity in the absence of any pathological condition (Robel et al., 2009; Kang et al., 2014). One important signal mediator involved in various signaling pathways, is the aforementioned lipid and protein phosphatase PTEN, a key molecule in antagonizing the PI3K signaling pathways (Kreis et al., 2014). PTEN is involved in the regulation of cell size and proliferation of astrocytes as well as the formation of glial scars (Fraser et al., 2004; Dey et al., 2008; Renault-Mihara et al., 2017). Mechanistic details from the nestin-STAT3^+/–^ mouse model which has impaired astrogliosis show that the affected astrocytes upregulate PTEN. The intrinsic defects in glial scarring comprising process orientation and elongation as well as leukocyte seclusion are rescued through PTEN inhibition, which evokes substantial cytoskeletal remodeling by changing the activation state of the small GTPases RhoA and its downstream effectors (Table 1; Renault-Mihara et al., 2017).

**Table 1:** Mouse disease models targeting or affecting cytoskeletal regulators *in vivo*.

Mouse models	Effects in disease models	Open questions and remarks
**Cdc42** Tamoxifen-induced *GLAST/eGFP Cdc42*^loxP/loxP^ mice Traumatic brain injury (Robel et al., 2011; Bardehle et al., 2013)	Impairment of pathology-induced proliferation of astrocytes but increased numbers in microglia upon traumatic brain injury. No obvious defects in microtubule organization and dynamics.	No process outgrowth related-phenotype in contrast to previously reported microtubule dependent defects in cultured astrocytes (Etienne-Manneville and Hall, 2001). Unclear, whether diverging outcomes rely on differences between cultured and astrocytes *in vivo*, or mosaic pattern of scattered Cdc42-deficient astrocytes *in vivo*.
**GFAP and Vimentin** *GFAP*^−/−^*Vim*^−/−^ mice Brain and spinal cord injuries (Pekny et al., 1999) Sciatic nerve lesion (Berg et al., 2013) Entorhinal cortex lesion (Wilhelmsson et al., 2004) Photothrombosis model for stroke (Liu et al., 2014) *GFAP*^−/−^*Vimentin*^−/−^*PPT1 *^−/−^ mice 5. Batten disease (Macauley et al., 2011) *GFAP*^−/−^*Vimentin*^−/−^ *in APPswe/PS1dE9 AD background* 6. Alzheimers disease (Kamphuis et al., 2014)	Impaired hypertrophy of cell bodies and main processes, less dense glial scars, frequent bleedings into lesion sites. Complete axon regeneration. Enhanced Synaptic regeneration. Enhanced axonal remodeling and improved motor recovery. Rapid onset and progression of disease through impaired blood-brain-barrier and profound neuroinflammatory response. Less interaction of astrocytes with Aβ plaques. No effect on Aβ plaque load.	Wide range in beneficial and detrimental phenotypes by GFAP/Vimentin deficient mice among the different disease models.
**STAT3** Nestin-Stat3^−/−^ Mice Spinal cord injury (Renault-Mihara et al., 2017)	Attenuated up-regulation of GFAP, failure of astrocyte hypertrophy, and pronounced disruption of astroglial scar. Reactive astrocytes fail to elongate and show no preferential orientation towards the lesion. Reduced activation of RhoA. Rescuable by reduction in PTEN.	Study indicates a predominant role of the actin cytoskeleton in reactive astrocytes in contrast to previous findings (Etienne-Manneville and Hall, 2001).
**Palladin** Adult rats Cerebral cortex injury (Boukhelifa et al., 2003)	Upregulation of Palladin.	Role in modulation of actin cytoskeleton upon injury not explored.
**a-Actinin** Adult mice Cortical stab wound injury (Abd-El-Basset and Fedoroff, 1997)	Upregulation of a-actinin.	Role in modulation of actin cytoskeleton upon injury not explored.

*Summary of in vivo studies with mouse disease models, which, either directly addressed or reported phenotypes associated with the cytoskeleton*.

In contrast to the wealth of knowledge on stimuli, receptors and signaling pathways evoking astrogliosis, we know very little about the downstream machinery exerting the functional and morphological changes in reactive astrocytes. One factor partially responsible for the swelling of astrocytes is simply water, which is increasingly taken up through aquaporin-4, and which reactive astrocytes upregulate and redistribute from their perivascular endfeet over the entire plasma membrane (Saadoun et al., 2005; Ren et al., 2013). Aquaporin-4-dependent water uptake contributes to the defense response of astrocytes, as shown in loss of function experiments, where deleting this water channel perturbs the ability of astrocytes to migrate in cell culture and to form glial scars *in vivo* (Saadoun et al., 2005). Furthermore, loss of aquaporin-4 impairs astrocyte secretion of proinflammatory cytokines in autoimmune encephalitis (Liu et al., 2014). However, the effective impact of aquaporin-4-dependent water uptake in reactive astrocytes in pathological conditions is currently under debate. Depending on the individual nature of the pathological condition, changes in astrocytic aquaporin-4 can either facilitate or counteract the formation of cerebral edema (for a review see Stokum et al., 2015).

Another major driving force in hypertrophy and process outgrowth in scar-forming astrocytes is the cytoskeleton. Below we discuss in depth the individual filament systems of the cytoskeleton in reactive astrocytes and their shape changes under pathological changes, and review *in vivo* analyses and studies using cultured astrocytes.

### Microtubule Activity in Scar-Forming Astrocytes

**Figure 5:**
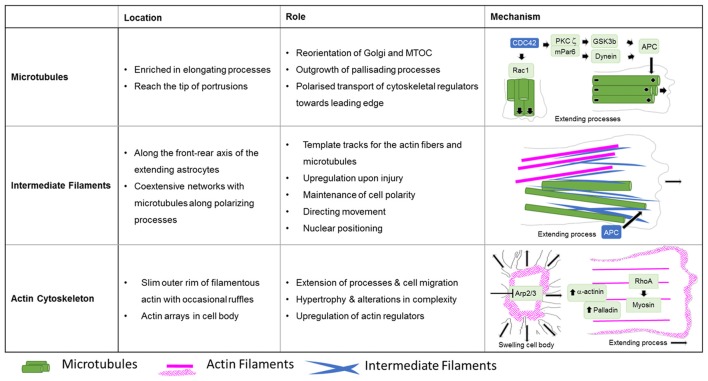
Roles of cytoskeletal systems during the Injury response of astrocytes *in vitro*. Summary in view of the localization and roles of microtubules, intermediate and actin filaments in cultured astrocytes upon injury. The schematic drawings depict the cytoskeletal systems and their major regulators involved in extension of astrocyte processes and cell body hypertrophy.

**Figure 6:**
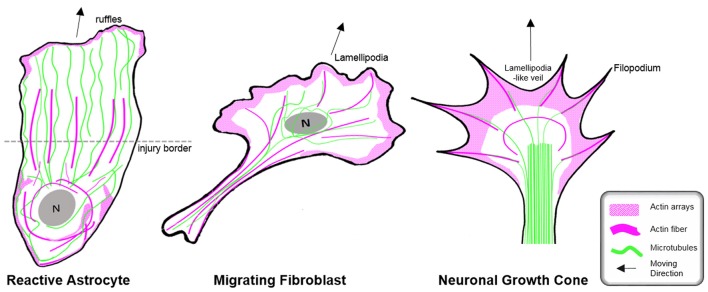
Organization of the cytoskeleton in reactive astrocytes in comparison to fibroblasts and neurons *in vitro*. Schematic drawings of microtubules (green) and F-actin arrays and fibers (magenta) in cultured mouse astrocytes after injury (left), migrating fibroblasts (center) and in extending axonal growth cones of developing neurons (right). The dashed line indicates the injury border and the nucleus is shown in gray (N). Elongated polygonal astrocytes at the wound edge extend into the cell-free space and have actin filaments (magenta) that are organized around the cell body and in bundles reaching into the center of the processes, while the leading edge possesses only a narrow outer rim of filamentous actin within sparse ruffles (left). Microtubules are enriched throughout the processes and reach into the extreme tip. In contrast, both fibroblasts and neuronal growth cones exhibit prominent actin arrays and fibers in the periphery of their leading edge forming filopodia, lamellipodia or lamellipodia-like veils. Most of their microtubules are present behind the actin-rich periphery, where microtubules form dense parallel bundles in growth cones. Microtubules extending into the actin-rich periphery frequently associate with actin fibers of filopodia.

Cell biology experiments provide substantial insights into the function of the microtubule network during palisading of scar-forming astrocytes. One of the most frequently used cell culture models is the scratch injury model of polygonal astrocytes grown in a 2D monolayer (Etienne-Manneville, 2006). This assay provokes the coordinated orientation, polarization and extension of astrocytes into the wound area, analogous to palisading astroglia during traumatic brain injury (Figures 5, 6), although it should be noted that cultured cells migrate unlike their counterparts *in vivo* (Bardehle et al., 2013). Astrocyte polarization begins with the microtubule-dependent reorientation of both the microtubule-organizing center and the Golgi apparatus (Etienne-Manneville and Hall, 2001). The reorientation of the Golgi in astrocytes by microtubules is distinct from that in other cells, as Golgi alignment relies solely on the actin cytoskeleton during migration of most other cell types (Magdalena et al., 2003). Microtubules are particularly enriched in long astrocytic processes (up to 150 μm; Figure 6). Moreover, microtubules reach the extreme tip of these astrocyte protrusions in contrast to other cell types, where only a minority of microtubules selectively enter the actin-enriched leading edge (Schober et al., 2007; Dent et al., 2011; Sakamoto et al., 2013). Treatment with the tubulin depolymerizing agent nocodazole demonstrated the integral function of microtubules for the directed outgrowth of palisade-like processes.

A key molecule in the directed elongation of astrocyte processes is the small GTPase Cdc42, which regulates the alignment of the microtubule-organizing center and Golgi through an mPar6-PKCzeta signaling complex (Etienne-Manneville and Hall, 2001). Downstream targets of Cdc42-mPar6-PKCzeta are dynein motor proteins and inactive GSK3-beta, which induces the steering interaction of the tumor suppressor protein, adenomatous polyposis coli (APC) with the microtubule plus-ends (Figure 5; Etienne-Manneville and Hall, 2003). Cdc42 is actively delivered towards the leading edge through anterograde transport by Arf6-positive vesicles (Osmani et al., 2010). Moreover, Cdc42 controls microtubule-dependent outgrowth of astrocyte processes through Rac1 (Figure 5; Etienne-Manneville and Hall, 2001). However, inducible genetic deletion of Cdc42 *in vivo* does not affect the orientation of palisading astrocytes but impairs their pathology-induced proliferation in traumatic brain injuries (Table 1; Robel et al., 2011; Bardehle et al., 2013). Whether this *in vivo* phenotype relies on differences in microtubule regulation between genuine and cultured astrocytes or the mosaic pattern of scattered Cdc42 deficient astrocytes influenced by the majority of surrounding wildtype cells, is currently unknown. Future work will need to further study the significance of microtubules in reactive astrocytes through comprehensive studies *in vivo* and additional cell culture models.

### Intermediate Filaments in Glial Scarring

Because of the prominent upregulation of intermediate proteins in reactive astrocytes, this filament network is traditionally seen as the most relevant cytoskeletal system in astrogliosis (Sultana et al., 2000). In contrast to during development, intermediate filaments substantially participate in astrogliosis and glial scarring, with their impact dependent on the CNS region and the type of pathology. Of note, GFAP and vimentin deficiency impairs the hypertrophy of cell bodies and main processes in reactive astrocytes. Moreover, glial scars that occur as a consequence of brain or spinal cord injuries are less dense and are frequently accompanied by significant bleeding in the central lesion sites (Table 1; Pekny et al., 1999). Deleting both intermediate proteins affects axon remodeling and motor behavioral recovery in mice after stroke and correlates with increased sensitivity of astrocytes to oxidative stress during hypoxia and subsequent reperfusion (de Pablo et al., 2013; Liu et al., 2014). Furthermore, the loss of GFAP and vimentin in a mouse model for chronic neurodegenerative Batten disease impairs the blood-brain barrier and accelerates the onset and progression of the pathology (Macauley et al., 2011). The absence of intermediate filaments also affects the interaction of astrocytes with amyloid plaques in AD mouse models, albeit without significant effect on the AD plaque load (Table 1; Kamphuis et al., 2014). However, deficiency in GFAP and vimentin may also have beneficial effects, as shown in GFAP- and vimentin-deficient mice exhibiting complete axon regeneration after induction of a sciatic nerve lesion (Table 1; Berg et al., 2013). In addition, a lack of GFAP and vimentin induces remarkable synaptic regeneration after entorhinal cortex lesions (Table 1; Wilhelmsson et al., 2004).

The fine structure of the intermediate filament network was mainly studied in culture after scratch induced-injuries, where intermediate filaments run along the cells’ front-rear polarity axis in elongating astrocytes (Sakamoto et al., 2013). Intermediate filaments play an important role in establishing and maintaining cell polarity, directing movement and control of nuclear positioning, and interacting and coordinating with other filament systems of the cytoskeleton (Dupin et al., 2011; Sakamoto et al., 2013; Leduc and Etienne-Manneville, 2017). Intermediate filaments and microtubules create coextensive networks along the elongated processes of astrocytes (Figure 5) and the arrangement of both filament types is coordinated through APC (Sakamoto et al., 2013). On the one hand, a major role of intermediate filaments may involve the creation of template tracks for microtubules to enhance persistence of cell polarity and directed movement (Figure 5; Gan et al., 2016). Alternatively, microtubules may be required to establish stabilizing intermediate filaments according to increased anterograde and reduced retrograde transport of intermediate filament subunits along microtubules in extending astrocytes (Leduc and Etienne-Manneville, 2017). Along with microtubules, intermediate filaments associate with actin filaments and control the assembly of actin fibers and orientate traction forces for direct cell movement (Figure 5; Costigliola et al., 2017; Jiu et al., 2017).

### The Actin Cytoskeleton During Astrogliosis

In contrast to the comprehensive knowledge on the actin cytoskeleton in non-neuronal cells and neurons, little is currently known regarding the nature and function of the actin filament cytoskeleton in astrocytes in general (as described above) and, in particular, during astrogliosis. Available data indicate distinct differences in the actin organization of polarizing astrocytes after injury, compared to other extending or migrating cells (Figure 6; Dent et al., 2011; Steffen et al., 2017). In cell culture, protruding processes of polygonal astrocytes exhibit a slim outer rim of filamentous actin with occasional ruffles, where other growing or migrating cells such as fibroblasts and neurons possess prominent actin meshworks and fibers in their periphery and form lamellipodia and filopodia (Figure 6; Etienne-Manneville and Hall, 2001). In scar-forming astrocytes, most actin filaments are instead concentrated around the cell body. Depolymerizing actin filaments with the toxin cytochalasin D seems to have little effect on the formation of long processes in polygonal astrocytes but blocks their artificial migration in scratch wound assays (Etienne-Manneville and Hall, 2001). In contrast, astrocytes with STAT3-dependent defects in reactivity and process elongation also present defects in actin-dependent focal adhesion disassembly and have substantially enhanced actomyosin tonus, while the organization of microtubules is unaffected (Table 1; Renault-Mihara et al., 2017). Moreover, astrocytes do respond to injury *in vitro* and *in vivo* by a drastic upregulation of the actin regulators α-actinin and its ligand palladin, which are known to reorganize actin filaments through crosslinking and bundling (Table 1; Abd-El-Basset and Fedoroff, 1997; Boukhelifa et al., 2003). The particular injury-specific increase and accumulation of actin regulators in scar-forming astrocytes imply a currently unknown contribution of the actin cytoskeleton to glial scarring.

Although the relevance of actin dynamics in palisading astrocytes is not clear, studies in astrocytes in culture and in tissues indicate a role of the actin cytoskeleton in controlling hypertrophy and alterations in astrocyte complexity. Acute inhibition of the Arp2/3 complex in brain slices increases the astrocyte cell body size and abundance of large processes analogous to reactive astrocytes in diffuse trauma. Moreover, inactivating the Arp2/3 complex accelerates the hypertrophy of stellate astrocytes in the oxygen/glucose-deprivation model of stroke, whereas overactivating Arp2/3 by depleting its endogenous inhibitor PICK1 or overexpressing the activator N-WASP suppresses cell body expansion (Figure 5; Murk et al., 2013). This is in line with other studies indicating a switch from Rac1 and Arp2/3-dependent networks towards actin organization in reactive astrocytes relying on RhoA and linear actin filaments in association with myosin (John, 2004; Renault-Mihara et al., 2017). However, the expansion of astrocyte cell bodies and main processes upon Arp2/3 inhibition is another indication of distinct actin organization in astrocytes compared to other cells, where the Arp2/3 inactivation instead leads to shrinkage, collapse or inhibited outgrowth (Figure 6; Korobova and Svitkina, 2008; Wu et al., 2012).

## Concluding Remarks

The morphological features of astrocytes are integral to the normal shape and functioning of the CNS from late embryonic development throughout all stages of an organism’s life. Despite their critical relevance in both normal and disease states, the molecular mechanisms behind the plastic morphology of astrocytes are poorly understood. Nonetheless, improvements in cell culture methods and the development of new tools and elaboration of sophisticated microscopy techniques will allow observation and manipulation of more authentic astrocyte settings. In this context, nanoscopic PAP-synapse interactions are becoming increasingly accessible with super-resolution light microscopy, and as such, provide an appealing experimental alternative to sophisticated electron microscopy (Heller et al., 2017). Recently introduced astrocyte-specific and inducible gene targeting models will allow the functional characterization of astrocytes throughout the entire CNS without undesired collateral damage in neurogenic radial glia cells (Srinivasan et al., 2016; Winchenbach et al., 2016). Finally, human-specific and disease-relevant characteristics of astrocytes, which are associated with abnormal morphologies and functions (Windrem et al., 2017) can now be studied *in vivo*. The substitution of murine astrocytes through the implantation of glial progenitors derived from human-iPSC makes it possible to investigate the specific role and contribution of abnormal astrocytes in diverse pathologies. A better understanding of the plasticity of astrocyte morphology and function will help us to gain insight into the fundamental properties of this profound shapeshifter in both normal cells and diverse human pathologies.

## Author Contributions

JS, BJE and KM wrote the manuscript. JS and KM created the figures.

## Conflict of Interest Statement

The authors declare that the research was conducted in the absence of any commercial or financial relationships that could be construed as a potential conflict of interest.
